# Difference in factors associated with low-level viraemia and virological failure: results from the Austrian HIV Cohort Study

**DOI:** 10.7448/IAS.17.4.19667

**Published:** 2014-11-02

**Authors:** Gisela Leierer, Armin Rieger, Andrea Steuer, Mario Sarcletti, Maria Geit, Bernhard Haas, Ninon Taylor, Manfred Kanatschnig, Michaela Rappold, Bruno Ledergerber, Robert Zangerle

**Affiliations:** 1 Department of Dermatology and Venereology, Innsbruck Medical University, Innsbruck, Austria; 2 Department of Dermatology, Medical University Vienna, Vienna, Austria; 3 Department of Pulmonary Medicine, Otto Wagner Hospital, Vienna, Austria; 4 Department of Dermatology, Allgemeines Krankenhaus Linz, Linz, Austria; 5 Department of Infectious Diseases, Landeskrankenhaus Graz West, Graz, Austria; 6 Department of Internal Medicine III, Paracelsus Medical University Salzburg, Salzburg, Austria; 7 Department of Internal Medicine, Landeskrankenhaus Klagenfurt, Klagenfurt, Austria; 8 Division of Infectious Diseases, University Hospital Zurich, Zurich, Austria

## Abstract

**Introduction:**

For some patients, it remains a challenge to achieve complete virological suppression which is the goal of antiretroviral therapy (ART). Identifying factors associated with low-level viraemia (LLV) and virological failure (VF) under ART might help to optimize management of these patients.

**Materials and Methods:**

We investigated patients from the Austrian HIV Cohort Study receiving unmodified ART for >6 months with two nucleoside reverse-transcriptase inhibitors (NRTIs) with either a non-nucleoside reverse-transcriptase inhibitor (NNRTI) or a boosted protease inhibitor (PI) or an integrase inhibitor (INSTI) between 1 July 2012 and 1 July 2013 with at least one viral load (VL) measurement below the limit of detection (BLD) or below level of quantification (BLQ) in their treatment history. VF was defined as HIV-RNA levels ≥200 copies/mL and all other quantifiable measurements were classified as LLV. Factors associated with LLV and VF compared to BLD and BLQ were identified by using logistic regression models.

**Results:**

Of the 2,276 patients analyzed, 1,972 (86.6%) were BLD or BLQ, 222 (9.8%) showed LLV and 82 (3.6%) had VF. A higher risk for LLV and VF was found in patients with ART interruptions and in patients with boosted PI therapy. The risk for LLV and VF was lower in patients from a centre which uses Abbott RealTime HIV-1 assay compared to the other centres measuring VL by the Roche Cobas AmpliPrep/Cobas TaqMan 2.0. A higher risk for LLV but not for VF was found in patients with a higher VL before ART and shorter ART duration. A higher risk for VF but not for LLV was found in patients of younger age, originating from a high prevalence country, with a lower CD4 count and in male injecting drug users.

**Conclusions:**

This study of well-defined patients on stable ART over a period of more than six months gives insights into the different factors associated with LLV and VF. In patients with VF, factors associated with adherence play a prominent role, whereas in patients with LLV, the biology of viral replication comes additionally into effect. Despite its observational design, it has implications for patient management and forms the basis for future outcome studies.

**Table 1:** Multivariable logistic regression results: Association between different factors and low-level viraemia and virological failure compared to viral load below the limit of detection or below level of quantification

	LLV<200	VF≥200
Outcome	OR (95% CI)	OR (95% CI)
Age at VL measurement		
< 30 years	1.01 (0.58–1.76)	2.95 (1.07–8.18)
30–50 years	0.98 (0.70–1.36)	2.80 (1.28–6.10)
> 50 years	1.00 (Reference)	1.00 (Reference)
HIV transmission category		
Male injecting drug user	1.07 (0.66–1.73)	2.04 (1.00–4.18)
Female injecting drug user	0.42 (0.16–1.08)	1.44 (0.48–4.28)
Male heterosexual	0.85 (0.58–1.25)	0.69 (0.31–1.54)
Female heterosexual	0.62 (0.41–0.94)	1.11 (0.55–2.24)
Other	1.36 (0.72–2.58)	1.27 (0.35–4.61)
Men who have sex with men	1.00 (Reference)	1.00 (Reference)
Nationality		
High prevalence countries		2.14 (1.04–4.41)
Low prevalence countries		1.00 (Reference)
CD4 count at VL measurement		
Missing	2.12 (1.07–4.19)	0.65 (0.08–5.08)
< 200 cells/µL	1.70 (0.86–3.36)	9.17 (4.18–20.13)
200–349 cells/µL	0.98 (0.62–1.57)	2.81 (1.48–5.32)
350–499 cells/µL	0.94 (0.62–1.41)	2.53 (1.38–4.65)
≥ 500 cells/µL	1.00 (Reference)	1.00 (Reference)
Ever ART interruptions		
≥ 1	1.80 (1.33–2.43)	2.49 (1.53–4.05)
None	1.00 (Reference)	1.00 (Reference)
Assay used		
Abbott RealTime (single centre)	0.34 (0.21–0.53)	0.11 (0.03–0.35)
Roche TaqMan 2.0 (6 centres)	1.00 (Reference)	1.00 (Reference)
ART regimen		
2 NRTI + PI/r	1.53 (1.14–2.05)	2.35 (1.43–3.86)
2 NRTI + NNRTI/INSTI	1.00 (Reference)	1.00 (Reference)
VL before ART		
Missing	2.12 (1.03–4.37)	1.14 (0.42–3.07)
> 99.999 copies/mL	3.66 (1.92–7.00)	1.32 (0.59–2.98)
9.999–99.999 copies/mL	2.17 (1.11–4.22)	1.99 (0.90–4.41)
≤ 9.999 copies/mL	1.00 (Reference)	1.00 (Reference)
ART duration		
< 9 months	2.50 (1.33–4.72)	0.72 (0.16–3.24)
9–18 months	0.99 (0.57–1.71)	1.24 (0.55–2.79)
> 18 months	1.00 (Reference)	1.00 (Reference)

